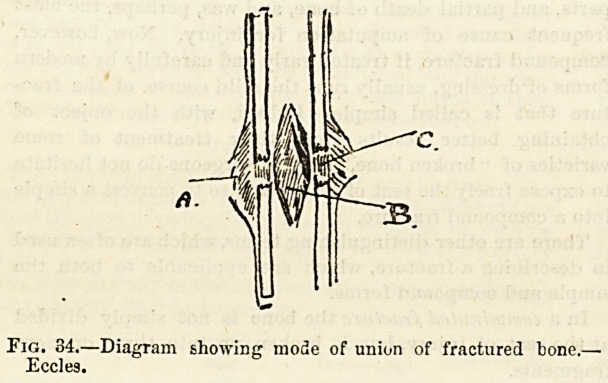# The Hospital. Nursing Section

**Published:** 1902-04-05

**Authors:** 


### The Hospital.

### Nursing Section.

Contributions for this Section of "The Hospital" should be addressed to the Editor, "The Hospital" Nursing Section, 28 & 29 Southampton Street, Strand, London, W.C.

No. 810.—Vol. XXXII. *SATURDAY, APRIL 5, 1902.*

### Notes on News from the Nursing World.

### QUEEN ALEXANDRA'S NURSING SERVICE.

The Royal Warrant issued last week brings into actual being Queen Alexandra's Imperial Military Nursing Service, and the old Army Nursing Service thus disappears. The interest of nurses generally will centre in the constitution of the Nursing Board, and especially in the appointments of the matron-in-chief and the two matrons of the civil hospitals. The appointment of Miss Browne as matron-in-chief is only of a temporary character. The civil hospitals could not be better represented on the board than by the matrons of St. Thomas's and King's College Hospitals. Miss Gordon is at the head of the great training school which will always be associated with Miss Nightingale's work, and has the confidence and the respect of the nursing world. Miss Monk is a born hospital matron, who has brought to bear upon her unwearied labours at King's a spirit of devotion to duty which is contagious, and has communicated itself to very many of the nurses who have been trained under her.

### MISS BROWNE'S CAREER.

Miss Sidney J. Browne, who has been appointed temporarily to the extremely important post of matron-in-chief of the Imperial Military Nursing Service, is now on her way home from South Africa. She was probationer at West Bromwich Hospital from 1879 to 1880, was staff nurse in the same institution for a year, and was at St. Bartholomew's Hospital from December, 1881, to April, 1882. On July 1st, 1883, she entered the Army Nursing Service at Netley, proceeding to Egypt in 1884. She returned the same year to Netley, but went again to Egypt in 1885, and served in the Nile campaign, receiving the medal and bronze star in recognition of her services. Subsequently she was appointed to the Herbert Hospital, Woolwich, where she remained until 1887, when she was nominated superintendent sister at the Military Hospital, Malta. In 1892 she was transferred to the Curragh Camp, and next became superintendent sister at the Connaught Hospital, Aldershot. Miss Browne has served in the South African campaign as superintendent of No. 3 General Hospital, and also of No. 12, and she has been awarded both the medal and the decoration of the Royal Red Cross.

### ROYALTY AND GUY'S HOSPITAL.

We understand that the Prince and Princess of Wales have graciously consented to open the new Nurses' Home at Guy's Hospital. No details have at present been settled, but it is not improbable that the event will be combined with the annual prize distribution.

### THE WAR NURSES.

Miss G. E. Styles, A.N.S.R., returned from South Africa on board the transport *Guelph*, arriving at Southampton last week. She requires one month's leave, and returns to South Africa. The *Manila* arrived at Southampton on the 27th, with the following nursing sisters on board:—M. Atkinson, A.N.S.R.; R. J. Briggs, A.N.S.R.; and G. E. Male, A.N.S.R. All require one month's leave, and return to South Africa.

### COMMEMORATING THE CORONATION IN CORNWALL.

It has been decided to mark the Coronation in the district of St. Teath by the establishment of a Nursing Association, and a committee has been elected for the purpose. The cost it is calculated, will be £50 a year at the outset, and an effort is to be made to raise this amount by means of three hundred subscriptions of 2s. a year, thirty of 10s., and the remaining £5 by collections in the seven places of worship in the parish. The promoters certainly do not take an unduly sanguine view of the liberality of the congregations at the seven places of worship.

### SALISBURY NURSES AND THE NATIONAL PENSION FUND.

At the annual meeting of the subscribers to the Salisbury Nurses' Home the report was adopted. It was stated that the affiliation of the institution to the Royal National Pension Fund for Nurses has worked very satisfactorily. Seventeen nurses have joined the scheme to secure an annuity of £15 at the age of 50. The sum paid by the Home amounted during the year to £163 15s. 4d. in addition to the sum paid by the nurses. The 25 nurses and seven probationers have nursed 260 cases, 26 of the patients being nursed gratuitously or at reduced rates. In future each probationer, on the completion of her term, is to take up duties as a resident nurse in the Home for at least six months. As Canon Warre stated, "during that time she will have the opportunity of further instruction in special matters in which her training in a public hospital has been deficient. Those who know the difficulties of a young nurse fresh from the hospital, sent for the first time into a large household, responsible for the charge of a valuable and perhaps precarious life, among strangers and strange associations, will realise how valuable this short period of quiet instruction and advice, after the rush and hurry of a hospital training, may be made in maturing the special qualities required in a first-rate nurse."

### THE PHYSICAL FITNESS OF PROBATIONERS.

The medical superintendent of the City Hospital, Little Bromwich, writes to protest against some comments in our issue of March 22nd, under the heading of "A Victim of a Lax System." We publish his letter gladly, but it in no way affects the issue. Dr. Beazeley says that the member of the nursing staff who died from typhoid was a probationer; but whether a girl of twenty-one is described as a nurse, or a probationer, does not affect the point. We did not suggest that the unfortunate young woman was either weakly or unhealthy, or that she was admitted without examination. Physical fitness, of course, includes age, and the fact that in this instance the probationer contracted typhoid is but a confirmation of our statement that "very young nurses are peculiarly susceptible." We trust that, in future, the question of age will be considered by the authorities of the Little Bromwich Hospital, as it ought to be considered in the case of all isolation hospitals, before probationers are admitted.

### HAMPSHIRE NURSES ASK FOR BADGES.

It is mentioned in the thirty-fourth annual report of the Hampshire Nurses' Institute that at the request of the nurses they have been provided with badges. The committee think that the badges may be an additional incentive to the faithful discharge of their duties Certainly the concession is not one that can be criticised unfavourably. We are glad to see that the record for the year was of a satisfactory character. The total amount of fees earned was £1,291, and the balance at the bank is £100 13s. 7d., a slight increase on that of last year.

### NURSING AT GUERNSEY.

We have some further information from Guernsey in respect to the supply of nurses in the island. So far as general nursing is concerned, it appears that there is one trained nurse attached to the Victoria Cottage Hospital who goes out to private cases, and one maternity nurse, working on her own account, who attends midwifery cases. These, in ordinary circumstances, are sufficient, but when emergencies arise they cannot be met. Residents or visitors suffering from an infectious disease must apparently be nursed at an isolation hospital or not at all. Happily, there has been a great improvement in the condition of things at the Town Isolation Hospital, under the auspices of the present matron. In addition to every attention from the medical superintendent, the patients have now the advantage of bright and home-like surroundings. But the Town Isolation Hospital was built for people who cannot afford to pay, and whatever may be urged in favour of the general principle that all cases of infectious diseases should be nursed at hospitals, the state of affairs which prevails in Guernsey is not satisfactory.

### KEEP BUSINESS LETTERS.

The recent action of Miss Lloyd Harris against the Executive Committee of the Brighton, Hove, and Preston District Nursing Association once more emphasises a point which we have repeatedly endeavoured to impress upon our readers, namely, that it is never safe to destroy any letters relative to business engagements as long as the arrangement is pending or proceeding. If nurses made this a golden rule they would seldom have to pay lawyer's fees, and would also escape a great deal of loss and trouble. Miss Harris stated that she arranged to become a probationer at the Sussex County Hospital, because the superintendent of the Nursing Association wrote to her that after two years' training she could become a Queen's Nurse. On the other hand, Miss Buckle, the superintendent, said she had advertised for a probationer to train for three years, and that no legal arrangements had been entered into for two years. The claim of Miss Harris was for £40 damages for loss of training promised and £8 8s. costs. But as neither side had kept the letters which passed, the matter was wisely settled out of court.

### THE "MIDDLE-CLASS" NURSE AT LEICESTER.

At the annual meeting of the Leicester and Leicestershire Institution of Trained Nurses, the Mayor urged the establishment of a group of nurses for the working class families who are ready and willing to pay for a trained nurse, but cannot afford the full fees charged to the well-to-do. The suggested introduction of a "middle-class nurse" was received with some favour, but no practical proposal was made in respect to it. It appears to us that the Leicester Institution has already as much on its hands as it can manage. If its sole object were to provide nurses for those who can afford to pay much or little, the question of charging reduced fees in certain cases would merit careful consideration. But as there is a district branch, we doubt the wisdom of trying to cater for yet another class. The idea that pecuniary support is not needed, which is fostered by the fact that the private nurses earned nearly £2,000 last year, would be strengthened by the announcement that another source of income had been tapped, to the detriment of the district branch. Thanks to special donations, the district branch had a balance on the right side at the end of the year, but even though in Leicester the two branches can be carried on successfully in conjunction, they would probably flourish still more apart.

### FREE TRAMWAY PASSES.

It was decided at the twelfth annual meeting of the Cardiff and District Branch of Queen Victoria's Jubilee Institute for Nurses that a request should be addressed to the Corporation of Cardiff asking them to grant free passes to the nurses of the institute over the tramways of the town. Dr. Paterson said that the difficulty was a legal one, but another speaker pointed out that free passes were granted to Jubilee nurses by the Corporations of Bolton, Sheffield, and Dundee. A vote of condolence with the widow of Mr. William Rathbone was passed. Owing to Mr. Rathbone's influence a Liverpool gentleman subscribed £100 to the funds of the Cardiff Institute.

### SMALL SUBSCRIPTIONS WANTED.

The scope of the work of the Sunderland District Nursing Association continues to extend. The number of patients nursed during the past year was 955, being 120 in excess of the previous year, while the number of visits paid were 26,366, an increase of 3,840. There are now eight nurses, as well as the superintendent, an increase of two since last year. The other side of the shield is not equally satisfactory, for, notwithstanding that much has been done, there is still a balance of £96 10s. 7d. due to the Bank. This is the less creditable to a flourishing town like Sunderland, because one lady has undertaken to entirely defray the cost of an extra nurse for a particular district, in addition to the £10 which she already subscribes to the ordinary fund. The patients contributed £63 2s. in thank offerings. The great want is chiefly more annual subscriptions of small sums.

### A "CHRONIC'S" REPROACH.

A district nurse in the East End of London recently contracted influenza. Owing to considerable pressure it was impossible to get her place filled at once, and the less serious cases had to stand over for a little time. Amongst these was an old "chronic" who had been on the books of the association for three years. When the nurse returned to her labours a few days later she was greeted reproachfully by the old dame. "Well, nurse, do you know it is a whole week since anyone has come near me?" The nurse explained the situation. "Yes, yes, but when my own nurse fell ill, two years ago, I was not left alone. Queen Victoria sent me another one down next day. But times has changed since then, and I expect the new Queen don't know me."

### THE NURSES' HOME AT GLOUCESTER INFIRMARY.

The Honorary President of the General Infirmary at Gloucester and the Gloucestershire Eye Institution and the Chairman of the General Committee have issued an appeal on behalf of the scheme recently propounded, the principal feature of which is the erection of a separate home for the nurses. This and other necessary improvements will, it is estimated, cost £10,000, and the first list of subscriptions shows a total contribution of £2,600 from 21 donors. The President, the Earl of Ducie, gives £500, Mrs. W. E. Price £300, Lord Fitzhardinge £200, and Earl Bathurst £100. There are 13 other subscriptions of £100 each.

### MUCH HADHAM NURSING ASSOCIATION.

The second annual meeting of the Much Hadham Nursing Association was held at Moor Place, on Friday, last week. The report of the Executive Committee and the accounts were laid before the meeting and adopted. During the year the nurse attended 83 cases, necessitating in all 2,559 separate visits. The accounts showed that the total expenses amounted to £110 15s. 1d., and the subscriptions and donations to £122 1s. 5d., leaving a balance in hand at the end of the year of £11 6s. 4d. The total number of subscribers was 183, of whom 121 subscribed 2s. 6d. each or less. A resolution was passed approving the report and accounts, and the valuable services of the nurse were acknowledged.

### HIS BROWN TONGUE.

A rather amusing story comes to us in regard to an incident which happened in the male ward of a hospital the other day. After re-applying the splints to a fractured leg, the surgeon told the patient to put out his tongue—which he did, whereupon the surgeon observed, "Not very good," and straightway prescribed a medicine to be taken three times a day; then, turning to the sister, he said, "It is through lying in bed and having plenty of good food." As soon as the doctor had left the ward, the man remarked, "I was eating chocolate when he came in, and daren't speak." This speech was greeted with a roar of laughter; the youth in the next bed saying, "I hope he's given 'im sumthin' nasty," and, judging from the conversation which followed, the affair was looked upon as a huge joke.

### MISCHIEVOUS INACTIVITY AT TRURO.

The Truro Board of Guardians have decided that the question of the revaccination of inmates of the workhouse and the nursing staff shall remain in abeyance. With praiseworthy foresight Dr. Salmon, the medical officer, had intimated that he proposed, on the first authenticated case of small-pox occurring in the county of Cornwall, to revaccinate all nurses and officers. This proposal the Guardians have declined to endorse, and in spite of the dire experience of the Mile End Guardians, we suppose that the same policy will be pursued at Truro. That is to say, unless or until small-pox appears in the county, the question of the revaccination of nurses will remain in abeyance.

### LIVERPOOL DISTRICT NURSES.

There were two features of exceptional interest in connection with the annual meeting of the Liverpool Queen Victoria District Nursing Association last week. Reference was made by several of the speakers to the support given by the late Mr. William Rathbone to the Association, the Lord Mayor and Monsignor Nugent in particular dwelling on the splendid help he afforded. In the report, of which the Lord Mayor moved the adoption, the council stated that in October last an appeal was privately circulated among the friends of the Association with the idea of inducing them to enable the council to assist and encourage nurses to subscribe to the Royal National Pension Fund for Nurses. Of the £4,000 asked for, upwards of £3,000 had, it is stated, been obtained, and a scheme was adopted. The Lord Mayor said he thought that the Pension Fund for Nurses was an excellent scheme, and it was generally commended.

### DISTRICT NURSING ASSOCIATIONS AND THE RATES.

There is a good deal of force in the contention that a district nurses' society is a rate-saving organisation. The ministrations of the district nurse tend to keep the home of the poor man together, directly by expediting the recovery of the breadwinner, and indirectly by often saving the children from being sent to the workhouse. Her labours not only supplement, but also diminish those of the relieving officer and the parish doctor. We are glad to see that this is how the matter is regarded in Bristol by some of the supporters of the admirable association which has just held its annual meeting, whose object is to provide a body of trained nurses to attend the sick poor particularly in their own homes. The report, unfortunately, shows a deficit of about £40, although, as every speaker at the meeting testified, the value of the work carried on by Miss Lloyd, the lady superintendent, and her staff, from every point of view, is beyond question. During the last twelve months, 3,269 cases were nursed, and 67,922 visits were paid, and several of the former were incurable cases at home, which could not be kept in the hospital. Dr. Myles mentioned that in his own experience he had known lives preserved through the instrumentality of the district nurses, and it was mentioned that in one parish many cases would be absolutely unattended if the association did not send one of its staff to visit them. With so many practical evidences of the usefulness of the society at their doors, the friendly societies and clubs, to whom special appeal was made, should gladly contribute enough to free it from debt, and to enable it to restore a nurse to the district from which the committee had to withdraw her in consequence of lack of means.

### Queen Alexandra's Imperial Military Nursing Service.

A Royal Warrant has been issued as a Special Army Order creating an Imperial Military Nursing Service, comprising the Army Nursing Service. The Nursing Board, under which this new corps is to be enlisted and controlled, is composed as follows:—

President—Her Majesty the Queen.

Vice-President—The Countess Roberts.

Chairmen—The Director-General, Army Medical Service, or, in his absence, Surgeon-General A. Keogh, C.B.

Two members of the Advisory Board—Sir F. Treves, K.C.V.O., C.B., and Major W. G. MacPherson, Royal Army Medical Corps.

Matron-in-Chief—Miss S. Browne (temporary).

Two matrons of civil hospitals—Miss Gordon and Miss Monk.

Representative of the India Office—To be named later by the Secretary of State for India.

Two members to be nominated by her Majesty—Viscountess Downe and Hon Sydney Holland.

The terms of the Special Army Order are as under:

Edward R.I.

Whereas we deem it expedient to further provide for the nursing services of our Army;

Our will and pleasure is that an Imperial Military Nursing Service, to be designated the "Queen Alexandra's Imperial Military Nursing Service," and comprising our "Army Nursing Service," shall be established, and the regulations contained in the warrant of our late Royal mother, dated October 26th, 1900, shall be amended as follows:

The Pay.

1. The following shall be inserted after Article 682:

682a. Appointments as nurses in our Queen Alexandra's Imperial Military Nursing Service shall be given to persons duly qualified under regulations approved by our Secretary of State.

682b. The pay of our Queen Alexandra's Imperial Military Nursing Service shall be as follows:

| | Initial rate. | Annual Increment. | Maximum. |
|---|---|---|---|
| Matron-in-Chief ... | £250 | £10 0 0 | £300 |
| Principal Matron ... | 150 | 5 0 0 | 180 |
| Matron ... ... | 70 | 5 0 0 | 120 |
| Sister ... ... | 37 10 0 | 2 10 0 | 50 |
| Nurse ... ... | 30 0 0 | 2 10 0 | 35 |

A matron may be granted charge pay at a rate not exceeding £30 a year, according to the magnitude of her charge.

682c. A member of the Army Nursing Service Reserve, called up for duty, shall receive pay at the rate of £40 a year. If appointed to a higher position than that of sister, she shall receive the pay of such higher grade.

682d. Pay may be issued in advance for a period not exceeding one month, prior to embarkation for service abroad.

682e. The pay of the female servant appointed to attend on the nursing sisters at Netley and Woolwich shall be £25 a year, and at other hospitals £15 a year, rising by annual increments of £1 to £20 a year.

682f. Pay during ordinary leave of absence may be granted in each financial year for the following periods:—

| | |
|---|---|
| Matron-in-chief ... ... ... ... | 6 weeks. |
| Principal matron ... ... ... | 6 " |
| Matron ... ... ... ... ... | 6 " |
| Sister ... ... ... ... ... | 5 " |
| Nurse ... ... ... ... ... | 4 " |

Pay may be granted for accumulated leave of absence during service at a station abroad.

682h. Pay during leave of absence on account of injury or sickness may be granted as under:

(*a*) When the injury or sickness is certified by the regulated medical authority to have been caused by the service, full pay may be issued for a period of twelve months, and half-pay for such further period as sick leave may be granted.

(*b*) When the injury or sickness is not caused by the service full pay may be granted for a period of three months; and after twenty years' service, two-thirds pay; or, with less than twenty years' service, half-pay for a further period of three months. In special circumstances, and subject to the approval of the General Officer Commanding, pay at the reduced rate may be granted for a third period of three months.

(*c*) When the sickness occurs at the station, a period not exceeding thirty days shall, if duly certified by the regulated medical authority, be excluded from the period of absence on ordinary leave to which the issue of pay is limited.

Terms of Retirement.

682i. Service as a nursing sister at a military hospital in the employ and pay of the National Aid Society, followed continuously by established service as a sister or nurse in Our Army Medical Service, may be allowed to count towards pension.

682j. A member of Our Queen Alexandra's Imperial Military Nursing Service may retire voluntarily on pension on attaining the age of fifty, and shall be compulsorily retired at the age of fifty-five.

682k. If pensioned on account of disability, one year of service in a tropical climate may count as two years towards pension.

682l. She shall be entitled to retire on pension after ten years' service if she is rendered unfit for hospital duty through disease or injury, certified by the regulated medical authority to have been caused by the service.

682m. She may at any time be required to retire on account of unfitness for the duties of her appointment, with such gratuity as she may be entitled to under Article 682r.

682n. The pension shall be calculated on the rate of pay at the time of retirement, and shall, after 10 years' service, be 30 per cent. of such pay, with an additional 2 per cent. for each year of service in excess of 10, up to a maximum of 70 per cent. of such pay.

In any case of special devotion to duty, a higher pension, not exceeding £50 a year, may be granted.

682p. If disabled in the service after five, but under 10, years' service, such rate of pension below that fixed in Article 682n shall be granted, as may be determined by Our Secretary of State. If she has served for less than five years when disabled, she shall receive a gratuity, to be determined in like manner.

682r. A member of Our Queen Alexandra's Imperial Military Nursing Service retired under Article 682m may, provided she has not been guilty of misconduct, be granted a gratuity of one month's pay for each year of service, if not entitled to a pension under Article 682n.

682s. In cases where a member of Our Queen Alexandra's Imperial Military Nursing Service is pensioned for a disability not permanently unfitting her for duty, the pension shall cease on the date when she again becomes fit for duty, unless there should then be no vacancy, in which case, should she be willing to continue her service, she may remain on pension for a period not exceeding one year, pending a vacancy.

682t. A member of Our Queen Alexandra's Imperial Military Nursing Service retiring without having previously obtained permission to do so shall forfeit all claim to pension or gratuity.

682v. A member of the Army Nursing Service Reserve who has been called up for duty shall, on the cessation of her employment, from causes beyond her own control, receive a gratuity of £20, provided that the principal medical officer under whom she has been employed certifies that she has rendered satisfactory service. If her employment has extended beyond one year she shall be granted, under the same conditions, a further gratuity, for each year or part of a year of further service, at the rate of £10 a year, if the service is given at home, and of £20 a year if given abroad. If a member of the reserve relinquishes her employment for reasons not satisfactory to our Secretary of State, she shall forfeit her title to a gratuity.

Article 371 and Articles 991 to 996c, 1,227 to 1,238a are hereby cancelled.

### QUEEN ALEXANDRA'S IMPERIAL MILITARY NURSING SERVICE.

Allowances.

It is our further will and pleasure that the following shall be substituted for paragraph 690 of the Regulations for the Allowances of our Army:

690. An allowance in lieu of board and washing at the rate of 15s. a week at a home station, or of 21s. a week at a station abroad, will be granted to each member of the Queen Alexandra's Imperial Military Nursing Service. A special allowance for the provision of clothing will also be granted, except to the matron-in-chief, at the following rates:

| | | |
|---|---|---|
| Annual clothing and cloak allowance | abroad | £9 0 0 |
| " " " | at home | £8 0 0 |

An allowance of 10s. 6d. a week for board, etc., will be granted to the servant appointed to attend on the members of the Queen Alexandra's Imperial Military Nursing Service. The other allowances at stations abroad, including the allowance for servants, will be at such rates, not exceeding those of a departmental officer of subaltern rank, as the Secretary of State may determine.

The provisions of this paragraph will apply to members of the Army Nursing Service Reserve when called up for duty.

Given at Our Court at St. James's this 27th day of March, 1902, in the 2nd year of Our Reign, by his Majesty's Command,

St. John Brodrick.

### Lectures to Nurses on Anatomy.

By W. Johnson Smith, F.R.C.S., Principal Medical Officer, Seamen's Hospital, Greenwich.

### LECTURE XIV.—GENERAL REMARKS ON FRACTURES OF BONES.

Having completed our review of the different parts of the human skeleton, let us, before passing to the study of other structures, regard our dry bones as living parts of a living body, and learn how they are influenced by and react to injury. It needs but a very brief experience of hospital work to convince us of the importance and interest attached to such an inquiry. Instances of "broken bone" are rarely absent from our casualty wards, and indeed form a majority of cases of injury.

*Fractures.*—There is probably not a single bone in the human body that may not be broken, although in some, as the sacrum, for instance, by reason of their inherent strength and protected position, and in others, like the small bones of the wrist, by reason of their small size, such injury is rarely met with.

Fracture is met with mostly amongst the labouring classes as the result of falls or violent blows, and occurs more frequently in males than in females. The bones most frequently broken in "middle-aged" persons are the ribs, the leg-bones, the thigh-bone, and the lower jaw. In infancy the bone that is most frequently broken is the collar-bone, in consequence of its being harder and less elastic than other parts of the skeleton at this period of life. In subjects of advanced age the most common fracture is that of the neck of the thigh-bone, which undergoes in senility changes in structure and direction, becoming more brittle and being set at almost a right angle to the shaft.

Fracture, though it may cause much inconvenience and more or less prolonged disablement, is not *in itself* a grave injury. A working man may, by an ordinary fracture of the leg or thigh, be prevented from supporting himself and his family for a period of two or three months, but, as a rule, his life is not endangered. The gravity of fracture lies in the *complications* of this injury, such as the laceration of soft parts, including blood-vessels and nerves, and the extent of contusion or bruising. Where the hard and resistent bone has yielded to force, the softer parts must also have given way. This is shown in cases of the serious and often fatal head injury known as "fracture of the base of the skull." The victim of such injury is in extreme danger, not because the cranial bones are cracked or broken, but on account of associated injury to the brain, nerves, and important blood-vessels.

The time-honoured distinction between *simple* and *compound* fractures has lost very much of its practical importance since the general adoption of antiseptic and aseptic methods in surgical work. We should, however, bear in mind the generally recognised meaning of these terms. *In a simple fracture*, however extensive and severe it may be in other respects, there is no wound of the skin, and, consequently, no way of ingress by which infective organisms can reach broken bone and bruised muscle. In a *compound fracture*, on the other hand, there is always a penetrating skin-wound, in some instances a mere puncture not much larger than the prick of a pin, in others an extensive and widely gaping gash. Before the days of Listerism a compound fracture almost always resulted in inflammation of the soft parts, and partial death of bone, and was, perhaps, the most frequent cause of amputation for injury. Now, however, compound fracture, if treated early and carefully by modern forms of dressing, usually runs the mild course of the fracture that is called simple. Indeed, with the object of obtaining better results from their treatment of some varieties of "broken bone," modern surgeons do not hesitate to expose freely the seat of injury and so to convert a simple into a compound fracture.

There are other distinguishing terms, which are often used in describing a fracture, which are applicable to both the simple and compound forms.

In a *comminuted fracture* the bone is not simply divided at the seat of injury, but is broken up into three or more fragments.

In *complicated fracture* the complication consists in the wounding of a large blood-vessel or nerve, or of some important organ—the lung, for instance, in fracture of one or more ribs; and the urinary bladder in fracture of the pelvis.

The terms *transverse*, *oblique*, and *spiral*, indicate the course taken by the line of fracture. Most fractures of long bones are more or less oblique; the spiral fracture is most frequently met with in the lower half of the tibia or shin bone.

*Linear*, *indented*, and *depressed fractures* occur in injuries to the bones of the skull. Linear fracture is a simple crack indented fracture is, as the term implies, a notch or pit produced on the surface of the bone; in depressed fracture, a common and very important variety of fracture of the skull, one portion of the broken cranial bone is depressed below the level of the rest.

In a "*green-stick*" or *incomplete fracture* one side only of the shaft of a long bone is broken, like as in a green stick bent across the knee. This injury occurs only in the pliant bones of young children, especially in the bones of the forearm.

The injury known as *separation of an epiphysis* is really another form of incomplete fracture. It occurs in young subjects in whom the layer of cartilage between the shaft and one end of a long bone—the epiphysial line—still exists (see Lecture XIII.). It is in most cases a fracture, as the line of division involves the bone and not the cartilage, and as the outer skin of the injured bone—the periosteum—remains intact and keeps the fragments in their natural position, this fracture may be regarded as incomplete.

### LECTURES TO NURSES ON ANATOMY.

The not very satisfactory term of *spontaneous fracture* is given to that form of injury in which a bone is broken by very slight force, or during a slight effort, as by a sudden movement of the body or a limb.

*Fracture by muscular action* is produced by violent or exaggerated muscular movements. The most frequent cause of fracture of the patella is sudden and very forcible contraction of the large mass of muscles in front of the thigh, but other bones are seldom broken in this way unless either the attached muscles act with unnatural violence as in an attack of convulsions, or the bone is weakened in consequence of disease. Next to the patella the humerus seems most liable to be thus injured. Instances are on record of this bone having been broken by a person striking at but not hitting another, by the gesticulations of a negro preacher, and by an effort to extract a tooth.

The process by which the fragments of a broken bone are *united* is a complicated and rather obscure one, in which not only the injured bone, but also the torn and contused muscles take place. The two main points to be comprehended are that the broken ends of the bone are first enclosed by a mass of soft and jelly-like tissue thrown out by the periosteum on the outer side (fig. 34 A), and the

Fig. 34.—Diagram showing mode of union of fractured bone.—Eccles.

medullary membrane on the inner side (B). This effused material becomes firm, and is converted either directly into bone or first into cartilage, and finally into bone, thus forming a natural splint. The ends of the bone are then directly joined together by a similar soft effusion which undergoes the same changes (C). As this direct bond of union which is called the *true or definitive callus* hardens, the external layer, called the *ensheathing or provisional callus*, and also the inner layer formed by the medullary membrane become absorbed.

The duration of this process varies in different bones, and is influenced by the age, the state of health and the constitutional vigour of the patient. In a healthy subject of middle age, the fractured bone, if in the upper extremity, should be firmly united by the end of the fourth week; if in the thigh or leg by the end of the second month.

In some few instances of fracture the fragments in consequence of wide separation or the interpolation of a thick layer of muscle, or, more frequently, as a result of some constitutional taint, do not unite, and a loose and flail-like junction or a so-called *false-joint* is produced. When this failure is persistent and very obstinate to surgical treatment it is classed as an instance of *ununited fracture*; when only temporary and simply an undue extension of the process of union, as one of *retarded union.*

In the treatment of fracture the surgeon endeavours in the first place to bring the fragments of the broken bone into their natural relations—*to reduce or set the fracture*; and in the next place to keep the fragments in good position until they have been joined together by callus. The first object he aims to effect by extension and manipulation of the injured limb, the separated fragments being thus pulled and pressed into position. The limb is then secured by bandages to suitable splints of wood or metal, or it is enclosed in some form of so-called fixed apparatus composed of bandages saturated with a semi-fluid substance which sets and forms a hard and closely-fitting appliance. The best known varieties of the fixed apparatus are: the splint devised by Mr. Croft, and that known as the Bavarian splint, both hardened by plaster of Paris; and the water-glass bandage. The chief advantage of appliances of this kind is that the period of confinement in bed shorten very much, and permit of what is called the *ambulatory treatment*, in which the patient is enabled to move on crutches from place to place.

*Diseases of Bone.*—The almost infinite variety of bone diseases may be roughly arranged into two large divisions: those of affections common to bones and other organs, and those of affections to which bones are more or less exclusively liable. Of the former, inflammation acute and chronic, tuberculosis, and cancer, are the most in evidence. The latter, of which the most common is rickets, form a long list of structural degenerations, of enlargements, of wastings, and of deformities, some due to acquired disease, some to inherited taint, and others to certain obscure influences of nerve disease.

### District Maternity Nursing in London.

By a Guy's Nurse.

Great are the difficulties to be overcome by the district maternity nurse in the poor parts of London, great also is the pleasure to be derived from the contemplation of the immediate and apparent effect of her work on the comfort of the patient, whom she has perhaps found in an almost hopeless chaos of pain and untidiness, and leaves tucked up in comparative comfort, in a bed as clean as circumstances will allow, with a sweet little morsel of pink humanity by her side.

For let no one dream that the babes that come to the poorest of the poor in the dirtiest parts of our great city are one whit less sweet and lovable than those that are born to fill delicate nests of silk and lace, prepared for them beforehand with every loving care, by the mothers of a more favoured class. Whatever they may become after a few months of neglect and bad feeding, and difficult as it may be to associate the poet's idea of "trailing clouds of glory" with the circumstances of their birth, the wee new-born babies of the slums are just as pretty in their innocence and unconsciousness as their more fortunate little brothers and sisters. The pity is that their tiny feet must so often learn to tread the ways of want and wickedness, and their little clinging fingers grow accustomed to the touch of coarseness, if not of cruelty.

### The Difficulties.

First and foremost among the difficulties is of course the fearful and wonderful amount of dirt to be encountered, in the form of matter very much "out of place," both dead, and alas! living. Very difficult is it to maintain the strict rules of asepsis, or even of antisepsis, when one is liable always to such trifling accidents as the slipping of the sooty and rimless lid of the family kettle into one's only available hot water, or the almost more disconcerting and sudden descent of various live creatures from their happy hunting-ground, the ceiling, into one's carefully prepared bowl of lotion!

### DISTRICT MATERNITY NURSING IN LONDON.

And, indeed, it is not always easy even to get the hot water in the first instance. It may involve fetching sticks to light the fire, or making a journey into a neighbour's premises to borrow a kettle, and it is not at all an uncommon thing to have to turn out the potatoes preparing for the family meal in order to procure a washing-basin for one's hands, and to displace the dripping meant for the children's tea to get a bowl for one's antiseptics. These things, of course, take time, and in some cases excite the strong disapprobation of the friendly neighbour, who, though perfectly willing to help her sister in trouble, is apt to regard the nurse's demands for hot water, and more hot water, as altogether extravagant and unnecessary.

The district nurse under such circumstances must learn to be an adept in the happy art of making the best (and the most) of things. She soon discovers how possible it is to do without almost everything she had previously deemed indispensable, and yet to arrive at happy results. Cleanliness, as far as her own actual work goes, she must have, and beyond that she can but hope that, use being second nature, the life lived in such surroundings will bring its own immunity from them.

### The Uncertainty.

Then, when all is for the present satisfactorily accomplished, there is always the feeling of uncertainty as to what may happen when one's back is turned, and the knowledge, even while one is exhorting to quietness and rest, that it is difficult for a mother with her family in the room around her and the last baby, perhaps scarcely able to toddle, to obtain very complete rest. But even so, things are often better than might be expected, for the little daughter, aged nine or ten, allowed by the School Board to stay at home in a case of emergency, will often prove herself a capable little person, both in looking after her small brothers and sisters and in scrubbing the floor and cooking the dinner.

### A Practical Illustration.

Never will those who had to do with her forget one dark-eyed little woman of nine, who acted as housekeeper, cook, and nurse-maid in one, during the unfortunately lengthened illness of the mother, and added to these functions those of part bread-winner, by selling collar-studs and other useful commodities in the streets when the "little" ones were in bed on a Saturday night!

### The Interest and Amusement.

And great also, if one has the wit to appreciate it, is the interest and the amusement to be derived from the many incidents, both comical and pathetic, with which one meets in this work and among these people. Many a mother is brave enough to while away the time of waiting with anecdotes and even jokes, and in all probability the maternity nurse learns more of the inner life and family history of the people she works among than any district visitor or clergyman would do. It is difficult, too, sometimes to know when to sympathise and when to scold, when to assist and when merely to preach thrift, in the face of plain evidence of money enough to get beer, but not enough to get baby clothes! She can give good advice which may or may not be acted upon, for the mother's argument usually is that she has had so many babies, and "buried" so large a proportion of them that she ought to know! But at any rate the nurse's advice is never resented, and the mother looks upon her as a friend, and is always proud to bring up the baby in all the glory of its christening robes, and present it for admiration with the remark, "This was your baby, Nuss; ain't it growed?" Poor little beings! they don't always grow. It is only too common a thing to meet the mother after some months, and on inquiry after baby to learn that he "wasted" at a few months old, and ended his little life in a fit of convulsions—small wonder, too, when probably gruel and sopped bread, and very likely cake and potatoes, formed part of his daily dietary.

### A Brighter Side.

But still there is a brighter side; some of the babies live and thrive, and some of the mothers take in what is taught them, and act upon it, and the district nurse's duty is clear, to impress upon them all, whether they will believe it or not, that nature's food alone, and where that is impossible the closest imitation of it, is what a baby requires. But drawbacks and disadvantages notwithstanding, it is a fact that for those who can put up with the sight of, and the contact with, much that is dirty and disagreeable, and who can appreciate, and to a certain extent admire, the cheerful-mindedness, and at least the good nature and fellow-helpfulness of the mothers of a very low degree, there are occupations which may be followed with less moral profitableness, and even less actual pleasure, than that of a maternity nurse in the slums of London.

### A Visit to the Foundling Hospital at Florence.

By a St. Bartholomew's Nurse.

When I was in Florence lately with a friend, who is also a nurse, we were struck by the decorations outside the Foundling Hospital, and after admiring Andrea della Robbia's quaint and beautiful medallions of swathed infants all along above the columns, we began to want to see how the living infants fared inside. We entered, asked the porter if we could see round, and were taken by him into the square. He then proceeded to call someone, in stentorian tones, and immediately a window opened and showed a sister's face surrounded by one of those curious caps with a waving piece each side. In a few moments she was at our side in the square, and asking us to follow her into a doorway. As we were not fluent in her native language, she agreed to say all she had to say to us in French; and very pleasantly she did it, never seeming to mind in the least if she had to repeat herself two or three times, as long as we understood in the end. She could not tell us the exact number of children then in hospital, for numbers are always changing; but about a hundred, more or less, is the average.

### Founded in the Fifteenth Century.

Each child, on admission, is weighed, and kept in a little ward to be seen by the doctor. I should say here that the hospital, when founded in the fifteenth century (the first of the kind in Europe), was intended solely for illegitimate children, but for some time now it has been possible for the very poor to have their babies taken care of here for the first twelve months of their existence. This must be a great boon to them, for they are cared for, as they could not possibly be in the wretched cottages from which some of them come. Though so old, the hospital is decidedly up to date. In restoring the building improvements have been introduced. There are no corners, all being rounded off in the most approved style.

### The Nursing.

The nurses, taken from the poorer classes; married women, acting where necessary as wet nurses, are dressed in holland—quite clean and neat, but lacking the smartness given by white cap, apron and cuffs. Each nurse is a mother to two babies, and sees to them day and night—during the

### A VISIT TO THE FOUNDLING HOSPITAL AT FLORENCE.

day in the nursery, and at night she has a little swinging cot on each side of her bed, in which her charges repose. Everywhere is spotlessly clean, and one sees no unnecessary furniture about. We found on inquiry that the cleaning of the institution is the work of former foundlings, some of whom return, and are employed in this way.

### The Nursery.

In the bedrooms are only the black iron bedsteads, with their white coverings, and on each side of them the tiny black and white cots to match. In the centre of the nursery is a large square table which has a raised edge, and is covered with something soft protected by jaconnette. On this, the babies are dressed and undressed very comfortably and easily. In the middle of the table is a metal urn-like arrangement containing boracic lotion, which is worked by the foot. Plenty of beautiful airing-cupboards were to be seen, as we flitted through the passages, and one had glimpses of shining copper baths, hot-water bottles, etc. Medicine and bandage cupboards were all bright and clean.

### The Sick Babies.

In the ward where the sick babies were, the chief thing to show us was a poor little hydrocephalic baby. The sister had the blinds drawn, and a lighted candle held behind the swollen head, which showed up the large gap between the sagittal sutures full of transparent fluid. Tongue-depressers and all such instruments are sterilised before use. The incubator is an interesting little room with its six tiny cots each having a square white shade over it. The babies seemed flourishing in their hot-house. The sister showed us an evil-looking spoon with a spout-like end for feeding by the nose when necessary. Infectious and specific diseases have their own quarters in the hospital. The tiny theatre, wards for sick nurses, and the large hall where every Florentine child is vaccinated free, we also saw.

### No Toys.

The bigger children have cots like those seen in children's hospitals in England; and in their nurseries they have mats to sprawl about upon, and little forms and tables. But no toys were visible when we went round; this may, however, have been because tea was expected shortly. In any case, the toddlers seemed quite happy gazing about them. When old enough the children are sent to cottages in the country, and a certain amount is paid to each cottager for keeping them. This sum diminishes as the child grows older and is able to do some work; and ceases for boys at the age of ten and for girls at fourteen. When marrying with the consent of the board the girls have a dowry of 235.20 Italian lire (about £95).

### Female Nurses for Male Lunatics.

By a Medical Superintendent.

It may not be impossible to train a man into a good sick nurse, but assuredly it is a difficult process to carry out, and most of those who have tried to effect the metamorphosis are of opinion that it would be quicker to train ten women than one man. The difficulty is one that has always been felt in our large county asylums, and various means have been tried to smooth it away. In the infirmary wards married couples have been placed in charge assisted by male attendants, and in some cases, I believe, by female nurses. Sometimes success, at others non-success, has attended these efforts. It will have been seen from a paragraph which appeared in The Hospital for March 8th that a more extensive trial of female nurses has been made in the male wards of the New York State Hospital for the Insane, and it is said that the innovation has been a source of satisfaction to all concerned in the institution. We are told that seven out of the ten wards have female nurses attached to them, but it does not seem certain whether these wards are entirely managed by female nurses or whether the nurses are merely additional to the usual staff of male attendants. If the former, it is manifest that some advantages might follow from the innovation, always provided that due selection were made in the class of patients in the wards, in the male attendants, and in the female nurses. To carry out all these provisos in seven-tenths of the wards of our county asylums would, I fear, be about as difficult as to train men into nurses. Anyone conversant with the inner life of our asylums will see how large these difficulties would loom if preparation were being made to inaugurate the American system in England. He would think of the construction of our wards as being unsuitable for a mixed staff; he would hardly know whether to place the nurses under control of the head nurse or the head attendant; he would hesitate before relegating duties to women which he had hitherto believed could only be properly performed by men; and he would remember that excepting in the infirmary wards not many of the duties which usually have to be done by men are, strictly speaking, "sick nursing." In fact, the practice was tried and found wanting in one of our county asylums about a quarter of a century since, and it is now almost confined to the sick wards of our asylums. Nevertheless, one failure is insufficient to prove the case, and mere theoretical objections cannot stand long against practical experience showing success. It was a maxim of one of the most celebrated medical superintendents in Great Britain that no lunatic should ever be forcibly fed until, if a man, the matron had been sent to feed him; and, if a woman, until the medical officer had tried his powers of persuasion. I feel sure that is a maxim which should be universally acted on.

There is a fairly well-grounded opinion that our county asylums are, taken as a whole, the best public hospitals for the insane in the world. New idols are ever being modelled and burnished in them. This latest innovation, if it be an innovation, will not pass unnoticed or untried; and results of these trials would be of public interest if the medical officers would send the requisite particulars.

### Death in Our Ranks.

We regret to hear of the death at Rawal Pindi, Punjab, India, on Sunday, March 2nd, of Sister B. L. Cann, of the Indian Army Nursing Service. She had been in an unsatisfactory state of health for some time, and shortly before had been invalided out of the service by a medical board. She was to have sailed in the *Hardinge* on the 15th inst. The immediate cause of death was peritonitis. There was a military funeral on the following day, and the band of "The Queen's" regiment was present.

Miss Hannah Arnold died at Bournemouth on March 18th of consumption, after a long illness. Miss Arnold was trained at King's College Hospital, London, and Chichester Infirmary. She was a private nurse at the Sarah Acland Home, Oxford, for three years, and had since been a member of the London Association of Nurses, 123 New Bond Street, for 14 years.

### The Queen at the Alexandra Hospital.

By a Correspondent.

The Queen, accompanied by Princess Victoria, and attended by the Hon. Charlotte Knollys, paid a private visit on Sunday afternoon, March 23rd, to the Alexandra Hospital for Hip Diseases, Queen Square, Bloomsbury.

Her Majesty, who went through all the wards, manifested a deep interest in the treatment carried out in each case, addressing a few kindly and sympathetic words to the occupant of each cot.

The children were not at all awed by the presence of Her Majesty, who won all hearts by the graceful and winning charm of her words and manner of distributing the supply of toys, dolls, and sweetmeats with which she had thoughtfully provided herself: each child receiving from her hands some memento of the visit in the form of a toy, and in addition a box of sweetmeats.

One small boy of five years embraced the Queen as she bent over his cot, and gave her a most affectionate kiss; he thought it quite the right thing to do. The Queen seemed very pleased and returned the embrace, and later on asked his name. Her Majesty addressed several of the nurses when inquiring after the special treatment of the children.

In one ward the children sang a hymn, very sweetly; and in another they sang "Soldiers of the Queen." Her Majesty smiled pleasantly, saying: "Oh! you darlings, how prettily you sing."

Many of the bigger boys saluted her Majesty, who seemed much gratified by the action. In one case, where a very small baby was difficult to please with a toy, the Queen went to great trouble to find her one she would like.

Another small child of four years sang two or three little items very sweetly. Her Majesty kissed him, saying, "You are a darling and sing so prettily."

The Queen remarked how well the children were looking, and reminded the officials that she had always been the patroness of this hospital, and that it was by her wish it was called "The Alexandra."

The visit lasted more than an hour. Before leaving Her Majesty signed her name in the visitors' book. She expressed herself delighted with the bright and beautiful wards, the happy look of the children, and the well-cared for appearance of the hospital generally, her words being, "What beautiful, bright wards those are"—and again, "How beautiful everything is."

The Queen during her visit conversed with the lady superintendent for some time asking several particulars about the working of the hospital, and observed that it had given her much pleasure to have had time to make the visit before going abroad.

### Appointments.

Bridgewater Infirmary.—Miss B. Luden has been appointed sister. She was trained at the New Hospital for Women, London.

Cardiff Infirmary.—Miss Mary Bratt has been appointed matron's assistant. She was trained at the Hospital for Women, Wolverhampton, and has since been sister at Nottingham Infirmary, staff nurse at the Hospital for Women, Chelsea, night superintendent and sister-in-charge of the women's wards at Bolton Infirmary.

Colchester Hospital.—Miss L. A. Rideout has been appointed sister. She was trained for three years at Addenbrooke's Hospital, Cambridge, and has since been staff nurse at the Victoria Hospital for Children, Chelsea, and done private nursing in the Riviera. Miss E. J. Woodward has been appointed sister. She was trained at Guy's Hospital, London, where she has since done holiday duty as sister. For two years she was sister at the Royal Hants County Hospital, Winchester, and she has been engaged in private nursing in East Dulwich.

Devonport Workhouse Infirmary. — Miss Margaret Priestman has been appointed superintendent nurse. She was trained at West Derby Union Infirmary, and has since been charge nurse at Wimbledon Isolation Hospital.

Durham County Hospital.—Miss Ella Francis Deakin has been appointed matron. She was trained at Newcastle Royal Infirmary, where she was afterwards staff nurse and sister. She has since been matron for 3½ years of Dene House Private Hospital, and for upwards of five years matron of Monkwearmouth Hospital, Sunderland.

Park Hill Hospital, Liverpool.—Miss Kate McLean has been appointed charge nurse. She was trained for three years at Whiston Infirmary, near Prescot, and has since been staff nurse for four years at St. Helen's Sanatorium.

Passmore Edwards Hospital, East Ham.—Miss M. Flory has been appointed matron. She was trained at Pendlebury Hospital, Manchester, and has since been matron at the Diphtheric Hospital, East Ham.

Punjab Command.—Miss M. L. Hayes has been appointed to officiate as lady superintendent, in the place of Miss Loch, who has proceeded to England on six months' sick leave. She was trained at St. John's Home, and after being attached to the nursing staffs at Charing Cross and the Metropolitan Hospital, entered the Indian Nursing Service, in which she has for some time senior sister.

Royal Infirmary, Hull.—Miss F. Lauris Smith has been appointed night superintendent. She was trained at the Royal Infirmary, Hull, where she also took sister's duty for six months. For four years she was nurse at the Norwich Isolation and Brook Hospitals. Her last appointment was that of staff nurse at the Mansfield and Mansfield Woodhouse District Hospitals.

Royal National Hospital for Consumption, Ventnor.—Miss Moyse-Hopkins has been appointed night sister. She was trained at the Royal Infirmary, Bristol, where she held the post of out-patient sister for five years. She was then appointed matron of the Memorial Hospital, Almondsbury, and has since acted as sister at a private nursing home in Clifton.

Royal Portsmouth Hospital.—Miss A. E. Cragg has been appointed sister of the children's ward. She was trained at the North Staffordshire Infirmary, and was afterwards staff nurse at the Children's Hospital, Newcastle-on-Tyne; charge nurse of the male and female wards and night sister at the North Staffordshire Infirmary; and sister of the female medical wards at the Royal Victoria Hospital, Belfast. Miss A. West has been appointed sister of the women's surgical ward. She was trained at the Swansea General Hospital, where she was afterwards staff nurse. Miss E. M. Thompson has been appointed theatre sister. She was trained at Guy's Hospital and was afterwards staff nurse in "Bright" ward.

St. Helen's Borough Sanatorium.—Miss Effie Bamford has been appointed sister. She was trained at the Mill Road Infirmary, Liverpool, for three years, and was afterwards staff nurse at Brompton Hospital for two years.

St. Olave's Infirmary.—Miss Rosina E. Hutchinson has been appointed charge nurse. She was trained at the Uxbridge Joint Hospital, and at St. Olave's Infirmary. She has since been engaged in private nursing. She is a member of the Royal British Nurses' Association.

Tunbridge Wells General Hospital.—Miss K. M. Nicolas has been appointed sister. She was trained at Croydon Infirmary for three years, and was afterwards private nurse at San Remo, Italy. She was also charge nurse at the Seaman's Branch Hospital, Albert Docks, for six months.

### Everybody's Opinion.

[Correspondence on all subjects is invited, but we cannot in any way be responsible for the opinions expressed by our correspondents. No communication can be entertained if the name and address of the correspondent are not given as a guarantee of good faith, but not necessarily for publication. All correspondents should write on one side of the paper only.]

### A VICTIM OF A LAX SYSTEM.

"Dr. Beazeley, Medical Superintendent of the City Hospital, Little Bromwich, Birmingham," writes: Under the heading "A Victim of a Lax System," you published in your issue of March 22nd an article calculated to bring grave discredit on this institution, and I must ask you to remove this impression by stating the true facts of the case. The member of our nursing staff we unfortunately lost from typhoid was a Probationer. As regards the "physical fitness" of our probationers, no one is appointed unless she is perfectly strong and healthy, and, moreover, everyone has to produce an independent medical certificate to that effect before her application for admission to the hospital is considered. "Previous experience" is *not* usually considered necessary for probationers.

### SLEEPING IN A PATIENT'S ROOM.

"Inexperienced" writes: A question has been raised amongst the nurses of the institute to which I am attached as to whether, in the case of a private patient (male), where there is only one nurse to take the case, a nurse does right to sleep in a patient's room. I recently had such a case. The patient, an elderly man, just required an occasional drink in the night. I had my bed in his room, there being no room leading out of the patient's, and I used to get up and attend to him. On returning to the home, and being asked how I got on, I happened to say what I had been doing, at which the more experienced private nurses seemed very shocked, and said, "They would not dream of doing such a thing." What should I have done under the circumstances? There was no one to share the nursing duties with me. Perhaps you or some of your readers will tell me.

[It may perhaps be said that there are exceptions to every rule, but we are sure that it must indeed be under very exceptional circumstances that such an arrangement as is above described can be regarded as allowable.

Ed. Hospital.]

### SHUTTING THE DOOR ON ROMAN CATHOLIC NURSES.

"A Lover of Justice" writes: I see from your issue of March 22nd that injustice to Roman Catholics is again causing just indignation to members of that Church. Recently I received from a matron of a nursing home in Yorkshire a letter, asking me to recommend some nurses, as she was so badly in need of them. After recommending one, who happened to be a Roman Catholic, I received another communication from the matron, saying she was deeply grieved that the fact of my candidate being a Roman Catholic disqualified her for the post. A few months ago I myself missed a matron's post from a similar cause. I therefore naturally share the indignation of the West Derby Guardians who protested against a policy of exclusion. My experience in institutions is that an official, particularly a nurse, without principle or religion, stands a much better chance of success than a person of principle if she happens to profess the Roman Catholic faith. Why is this injustice? Surely in these days of enlightenment, when bigotry is supposed to be extinct, a Roman Catholic or member of any other Church qualified in the work for which she is a candidate, should stand an equal chance.

### Where to Go.

St. James's Hall.—The talented Russian violinist, M. Michel de Sicard, is giving recitals, on Thursday afternoons, April 3rd, 10th, and 17th, at 3 p.m.

### For Reading to the Sick.

### THE WAY, THE TRUTH, AND THE LIFE.

O Way, through Whom our souls draw near
To yon eternal home of peace,
Where perfect love shall cast out fear,
And earth's vain toil and wandering cease;
In strength or weakness may we see
Our heavenward path, O Lord, through Thee.

O Truth, before Whose shrine we bow,
Thou priceless pearl for all who seek,
To Thee our earliest strength we vow,
Thy love will bless the pure and meek;
When dreams or mists beguile our sight,
Turn Thou our darkness into light.

O Life, the well that ever flows
To slake the thirst of those that faint,
Thy power to bless what Seraph knows?
Thy joy supreme what words can paint?
In earth's last hour of fleeting breath
Be Thou our Conqueror over death.

O Light, O Way, O Truth, O Life,
O Jesus, born mankind to save,
Give Thou Thy peace in deadliest strife,
Shed Thou Thy calm on stormiest wave;
Be Thou our Hope, our Joy, our Dread,
Lord of the living and the dead.

*Plumptre.*

There is a thought never far distant from the minds of men, no matter how careless they be or how keenly they resent it. It is the thought of God, the inward conviction that over and above them there reigns One in whose hand they are, who placed them here, who made all that surrounds them, who holds the reins of their lives and fashions their lot. It comes to them for better or for worse, for the fall and for the resurrection of many, as an invitation and consolation to be rejected or welcomed; still it comes, still it abides.

Help me, O God, to see Thee labouring continually within me and without, with a wisdom that reaches from end to end, and disposeth all things sweetly; to see Thy hand in every incident, and to remember that each is a grace directed to my good.

Thus we are brought to the feet of God in joyful adoration, in lowly praise and deepest gratitude, and enabled to realise that God loves us with an everlasting love. Having made us, He cannot forget us, or cease to love us. He is sweet to all, and His tender mercies are over all His works. He does not change. As a father hath compassion on his children, so hath the Lord compassion on them that fear Him, for he knoweth our frame and remembereth that we are dust. He is near us at all times; in the storm, in the calm, in the sunshine, and in the cloud; He watches over those who are weak and in pain, and moves others to help them. He will not suffer one of His little ones to perish, but bids them cast their care on Him, for He has a care for them. And so our eyes should ever be turned to Him, who alone can feed and sustain us; trusting Him for all we need, for it will surely come and shall not be slack, full measure, pressed down and overflowing, shall be poured into our bosom, when He opens His hand and fills the hungry with good things.—*Anon.*

### Echoes from the Outside World.

### Movements of Royalty.

The King, who is spending his Easter holiday on the Royal yacht *Victoria and Albert*, has signified to the Lord Mayor of Cork that he will be pleased to give a cup of the value of £100 to the Royal Munster Yacht Club, to be competed for in an international yacht race, to be held in connection with the International Exhibition at Cork. The Munster Club, in conjunction with the Royal Cork Yacht Club, will now prepare for a great race in Cork Harbour. There is every likelihood of the German Emperor's yacht *Meteor*, the unbeaten *Columbia*, and *Shamrock II.* being among the competitors.

The Queen's journeys to her native land—where she is spending Easter—not only give her pleasure, rest and health, but they very frequently result in the introduction of some fresh scheme for the aid of her English subjects. The fact that it was entirely owing to her influence that the Finsen treatment for cancer was introduced into the London Hospital will be remembered, and this came about after one of Her Majesty's periodical visits to Denmark. In Pall Mall just now can be seen another instance of the Queen's thoughtful observation. In Copenhagen, as Her Majesty has noted, the cabmen use small portable trestles on which their horses can rest their nose-bags and thus eat in much greater comfort. She has accordingly had some trestles sent over to her and given them to certain of the cabdrivers in Pall Mall, with the hope that the system may extend, and the horses be benefited.

### South Africa.

Although the death of Mr. Cecil Rhodes was not unexpected, owing to the alarming nature of the bulletins for some days past, the announcement of the event at Muisenberg, near Capetown, on Thursday last week, was not the less received with widespread regret. Mr. Rhodes was last in England in January, and on his arrival in South Africa, his health, which had been apparently somewhat improved by a brief stay in Egypt, broke down, as it is now known, hopelessly. His illness was watched in many parts of the world with anxiety, and the King and Queen were among the illustrious personages who sent him telegrams expressing their sympathy and hope for his recovery. Cecil Rhodes, who was only 48, was the son of a Hertfordshire clergyman, and a younger member of a family of seven brothers, whom their father, in the days of their childhood, spoke of as the "seven angels of the seven churches." Contrary to his hopes, not one of them took orders. The son who became great in other spheres went to Natal, while he was in his teens, on account of his health, and started farming, but at the age of 18 he had made a fortune in the diamond fields at Kimberley. With health restored, and financial independence assured in the space of two years, he returned to England in order to complete his education at Oxford. Here, however, his health again became seriously affected, and he consulted a great chest specialist, who advised an immediate return to South Africa, and wrote against the case in his note-book, "Not six months to live." The specialist was wrong, and Mr. Rhodes lived not only to finish his studies at Oxford and pass his examination, but also to become, by common consent of friends and foes, the foremost man in South Africa. It is not the least striking fact about his remarkable career that though he annexed territory, built railways, and erected telegraph lines, he had practically no private life. His last words were, "So little done; so much to do. Good-bye, God bless you."

On Thursday last week the embalmed body of Lieutenant Beverley Webster, a young Canadian officer who died at one of Lady Dudley's homes for disabled officers, left these shores for interment in his native land. He was wounded in South Africa, suffered from enteric, and was finally invalided to this country. Since his arrival just before Christmas he was most carefully tended, and the Commander-in-Chief personally visited him. His only chance of recovery seemed to be to return to Canada; his passage was taken, and Lord Roberts promised to present him with the South African medal before he left. When, however, the end suddenly came the body was fetched by a relative in South London, and subsequently Lord Roberts drove to the suburb in order to give into the hands of the heart-broken mother, who had come from Canada in order to be near her son, the hard-earned decoration. This characteristic act of kindness, accompanied by words of sympathy on the part of the Commander-in-Chief to the mother, was warmly appreciated.

On Sunday a disastrous railway accident occurred near Barberton, close to the Swaziland border of the Transvaal, involving the death of no less than 39 non-commissioned officers and men, and the injury of between forty and fifty more. The unfortunate victims of the accident were Hampshire men, and were about to be relieved by the recently-formed company which landed in South Africa a week ago.

### A Soldier Statesman.

The death took place on Saturday of Lieut.-General Sir Andrew Clarke, Agent-General for Victoria. Sir Andrew, who was born at Southsea on July 27, 1824, had a most brilliant career, and achieved the unique distinction of winning renown as a soldier in the field, a military engineer, who planned and supervised the construction of some of the most important of our land defences, a Colonial legislator and minister, the pro-consul of a province which he saved from the anarchy of tribal quarrels, and one of the handy-men of the Foreign Office list, who could be relied on to take over, at a moment's notice, any difficult task, in the way of bringing energy, intelligence, and tact to bear upon an awkward situation that had unexpectedly arisen in whatsoever part of the globe. Among the numerous posts he held was that of Inspector-General of Fortifications, and he constructed defences of coaling stations from plans of his own. He was, earlier in his career, Governor of the Straits Settlements.

### Philanthropic.

At the annual meeting of the Royal Albert Orphan Asylum, Bagshot, an interesting question was raised. It was proposed to discontinue the admission of girls into the home and to remove those now resident because they decline afterwards to enter domestic service. The author of the proposition had been connected with the institution for many years, and he said that there had of late been a great falling off in the number of candidates for admission. This he believed was because the Board School education was as good as that given in the Orphan Asylum, and as it left the children free on Saturdays and after and before school hours, the mothers were able to send the girls out as step-cleaners, etc., so that their earnings helped the family exchequer. Therefore they kept them at home. A lady speaker practically suggested that the age for leaving should be raised to 16 rather than 15 as at present, in order that the girls should be really old enough to make useful servants, and if they gave no "kit" unless the girls had a situation to go to, there would, she suggested, be an inducement to become servants instead of sempstresses or barmaids. Ultimately it was decided to do nothing for 12 months, so that the 60 girls at present in the institution will, at any rate, remain where they are for that period.

### The New Academician.

On Thursday last week Mr. George Frampton, A.RA., was elected a member of the Royal Academy. The new R.A., who was born in 1860, and studied modelling under Mr. Frith at the Lambeth School of Art, was awarded the Royal Academy gold medal at the age of 27, with a foreign travelling studentship of £200. Three years prior to that, in 1884, he began contributing to the summer show at Burlington House, and has since been represented at the exhibition without a break.

### Bank Holiday.

Upwards of 24,000 persons took advantage of the King's kindness in throwing Windsor Castle open to the public on Easter Monday. Some idea of the manner in which the multitude spent the day may be judged by the fact that 120,000 persons visited the Alexandra Palace, the same number the British Museum and the Galleries, 103,000 the Crystal Palace, 70,000 Kew Gardens, 37,000 the Zoological Gardens, 24,000 Hampton Court, 24,000 the Tower of London, and nearly 15,000 the Victoria and Albert Museum.

### Notes and Queries.

**The Editor is always willing to answer in this column, without any fee, all reasonable questions, as soon as possible.**
**But the following rules must be carefully observed:—**
**1. Every communication must be accompanied by the name and address of the writer.**
**2. The question must always bear upon nursing, directly or indirectly.**
**If an answer is required by letter a fee of half-a-crown must be enclosed with the note containing the inquiry, and we cannot undertake to forward letters addressed to correspondents making inquiries. It is therefore requested that our readers will not enclose either a stamp or a stamped envelope.**

*Lecturer.*

(1) To whom ought I to apply for the position of lecturer under the County Council on home nursing? I am a fully-trained nurse, and accustomed to lecture to large classes of probationers.—*Poulton-le-Fylde.*

The secretary, Technical Education Board, London County Council, Spring Gardens, S.W.

*Post.*

(2) I am anxious to obtain a post as nurse in a boys' school with sanatorium attached. I am fully qualified. Would you kindly tell me where to apply?—*Anxious.*

Advertise in scholastic papers.

*Sweden.*

(3) Will you kindly inform me if a Scotch lady, a trained nurse, could obtain hospital nursing or any other kind of work in Sweden, and how to set about it?—*M. P.*

Apply to the Swedish Consul, 52 Pont Street, S.W.

*Probationer.*

(4) Will you kindly inform me what are the duties of a probationer-nurse in a general hospital?—*M. F.*

You will find these described in Miss Honnor Morten's "How to Become a Nurse," Scientific Press, price 2s. 6d.

I am anxious to be trained as hospital nurse; will my father's having been a carman stand in the way of my being accepted as probationer?—*C. C.*

In a few schools only. See "The Nursing Profession: How and Where to Train," and write fully to the matron of such institutions as seem suitable.

Is there any hospital in London which would train me as nurse at twenty years of age?—*M. E. C.*

You are too young for a general hospital. See lists of children's hospitals in "The Nursing Profession: How and Where to Train," and apply.

Do you think that there is any chance of a girl in her nineteenth year becoming probationer in a hospital?—*D. C.*

Not the slightest.

1. What constitutes a training school recognised by the Local Government Board? 2. *M. G.* has been a probationer in the fever ward of a cottage hospital, and the matron (untrained) promises her a certificate at the end of three years. Will the certificate be of any use in obtaining good appointments? And, if not, what must *M. G.* do in order to gain a proper certificate?

Several qualifications are necessary in order for a training school to obtain recognition by the Local Government Board; one is that the superintendent nurse must be fully qualified, and another that the medical officer resides in the institution. In all cases of doubt it is as well to write to the Medical Inspector for Poor Law Purposes, Local Government Board, Whitehall, S.W., and ask. 2. She should enter for a three years' training at a good nursing school as soon as possible.

I am 19, and anxious to become a nurse in a children's hospital. Do any hospitals take probationers so young?—*Anxious.*

See reply to *M. E. C.*

Would you kindly answer my question in next week's edition of THE HOSPITAL. I am very anxious to become a probationer in a hospital. I am 21, strong and healthy, but I have only an elementary education. I have a book entitled "The Nursing Profession, etc.," but am in doubt about my education.—*L. S.*

Read the replies to other probationers, and apply to the matrons of Poor Law infirmaries. You can improve your education by study.

*Home for Incurables.*

(5) Would you tell me of a home for incurables for a gentleman suffering from locomotor ataxia? He has been a civil engineer, and a small fee of about 10s. a week could be paid for him.—*Nurse W. C.*

The case might be eligible for the British Home for Incurables, office, 72 Cheapside, E.C.; The Royal Hospital for Incurables, office, 106 Queen Victoria Street; St. Peter's Home and Sisterhood, Mortimer Road, Kilburn, N.W., or St. Andrew's Hospital for Convalescents and Incurables, Clewer, Windsor.

Do you know of any home where an old gentleman, without any ailment, but incapable of earning his own living, could be received for a very small fee?—*A. L. H.*

You give too few particulars. See "Burdett's Hospitals and Charities," under "Orphanages, Homes, and Charities."

Can you kindly tell me of a home or hospital where an imbecile boy could be received? Are there any in Norfolk? The boy's parents are very poor.—*Nurse R. E.*

He might be eligible for the Earlswood Asylum for Idiots and Imbeciles, Redhill (office, 36 King William Street, London, E.C.), or for the Darenth Schools for Imbecile Children, Dartford, Kent. There are no institutions especially for imbecile children in Norfolk.

Will you kindly tell me if there are any holiday homes, or homes of rest, for nurses at any of the following seaside resorts:—Margate, Hastings, or Southsea?—*E. S.*

Protestant Home of Rest, 98 High Street, Hastings.

I should be glad if you can tell me of a home for incurables, where a male patient could be received for a small fee?—*A. P.*

It is impossible to even suggest a suitable institution without some particulars of the case. See list of charities for chronic and incurable cases in "Burdett's Hospitals and Charities."

*Hernia.*

(6) Will you kindly tell me if there is a hospital devoted to hernia in London?—*A. C. S.*

No; but cases of hernia are treated at all the larger hospitals Certain institutions also, such as the City of London Truss Society, the National Truss Society, and the Surgical Aid Society, pay special attention to the instrumental treatment of this disorder.

*Male Nurse.*

(7) Would you be so kind as to tell me where I could obtain a three years' training as a male nurse, earning at the same time a small salary? I am 25 years of age, and have been engaged in mental nursing for the past six years.—*H. F. S.*

You can get a two years' certificate from the National Hospital for the Paralysed and Epileptic, Queen's Square, Bloomsbury, W.C. The salary is £12 for the first year and more for the second. There is no three years' training in nursing open to men.

*Baby.*

(8) I am very anxious to have charge of a baby in my own home, but in spite of advertising and replying to advertisements I have had no reply. Can you advise me?—*C. I. F.*

You could call upon the superintendent nurses of the lying-in charities, taking your references with you.

*Book.*

(9) Will you kindly recommend me a book on infantile ailments, and tell me where to get it?—*Nurse W.*

"The Mother's Help and Guide to the Domestic Management of her Children," by P. Murray Braidwood, M.D. Scientific Press. Price 2s.

*X-Rays.*

(10) I have been undergoing the X-Ray treatment for a tumour in my chest. I find it has burned my face and tanned me very much, besides producing an unpleasant irritation. Of what could I have a mask made in order to prevent further discomfort to my face and throat?—*X. R.*

Consult your medical man.

*Massage Cases.*

(11) Can you tell me of any institution that supplies massage cases to private homes working for themselves?—*C. L.*

There are none that we know of.

**Standard Books of Reference.**

**"The Nursing Profession: How and Where to Train." 2s. net; post free 2s. 4d.**
**"Burdett's Official Nursing Directory." 3s. net; post free, 3s. 4d.**
**"Burdett's Hospitals and Charities." 5s.**
**"The Nurses' Dictionary of Medical Terms." 2s.**
**"Burdett's Series of Nursing Text-Books." 1s. each.**
**"A Handbook for Nurses." (Illustrated). 5s.**
**"Nursing: Its Theory and Practice." New Edition. 3s. 6d.**
**"Helps in Sickness and to Health." Fifteenth Thousand. 5s.**
**"The Physiological Feeding of Infants." 1s.**
**"The Physiological Nursery Chart." 1s.; post free, 1s. 3d.**
**"Hospital Expenditure: The Commissariat." 2s. 6d.**
**All these are published by the SCIENTIFIC PRESS, Ltd., and may be obtained through any bookseller or direct from the publishers, 28 and 29 Southampton Street, London, W.C.**

## Figures and Tables

**Fig. 34. f1:**